# A Study of Synergy of Combination of Eosin B with Chloroquine, Artemisinin, and Sulphadoxine-Pyrimethamine on *Plasmodium falciparum In Vitro* and *Plasmodium berghei In Vivo*

**DOI:** 10.1155/2020/3013701

**Published:** 2020-06-01

**Authors:** Sedigheh Sadeghi, Zarrintaj Valadkhani, Alireza Sadeghi Tafreshi, Mohammad Arjmand, Hossein Nahravanian, Farideh Vahabi, Neda Soori, Maryam Mohammadi, Masoomeh Meigooni, Zahra Zamani

**Affiliations:** ^1^Department of Biochemistry, Pasteur Institute of Iran, Tehran 1439933831, Iran; ^2^Department of Parasitology, Pasteur Institute of Iran, Tehran 1439933831, Iran; ^3^Department of Parasitology and Mycology, School of Medicine, Zanjan University of Medical Sciences, Zanjan 4515613191, Iran

## Abstract

**Methods:**

Drug assessment was carried out singly or in combination on *Plasmodium falciparum in vitro* using the candle jar method at three inhibitory concentrations. Percent parasitemia of live cells was obtained by microscopic counting. Peter's suppression test was carried out on mice infected with *Plasmodium berghei* after 3 administration of the drugs singly and in combination, and parasites were counted by microscopy for 10 days.

**Results:**

Synergy was exhibited by isobolograms of eosin B combined with artesunate and sulphadoxine-pyrimethamine with more than 10 fold reduction of all drugs *in vitro.* A good combination index was obtained with artesunate at 50% inibitory concentration with 3.4 nM eosin B and 1.7 nM artesunate in contrast to 124 nM eosin B and 7.6 nM artesunate singly. *In vivo* studies also showed a considerable lowering of the effective dose of eosin B 30 mg/kg: artesunate 3 mg/kg with 200 mg/kg eosin B and 60 mg/kg artesunate separately. Sulphadoxine-pyrimethamine seemed to have the greatest synergistic effect with a combination index of 0.007, but this could be due to it consisting of a combination of three drugs. Eosin B's combination index with chloroquine was fair, and *in vivo* tests too did not show as much competence as the other two drugs. *Conclusion and Interpretation*. It can be concluded that eosin B can be used in combination with antimalarial drugs with favorable results.

## 1. Introduction

A major obstacle in the eradication of malaria is the development of drug-resistant parasites [1, 2]. Resistance to the standard antimalarial drug chloroquine was first reported in South East Asian countries and South America since the 1950s, and it has now reached the stage that it is no longer recommended in any African country [3–5].

Studies of high levels of resistance to chloroquine made some countries to switch their first-line drugs to sulphadoxine-pyrimethamine (SP), but unfortunately, the parasite also developed resistance to this drug combination with treatment failure being reported in Africa, Asia, and South America. One of the best-regarded drugs is artemisinin and its analogs against chloroquine-resistant *Plasmodium falciparum* infections which the WHO promotes in combination as a standard treatment globally. This is because its monotherapy has led to resistance in some areas of the world like the Mekong region [6, 7]. The fundamental principle of the remedial dose is that it efficiently kills most of the parasites while the remaining is completely eliminated by the high concentration of the partner drug [8]. The rapid action and short elimination period (less than one hour) of the action of artemisinin protect against a selection of mutant parasites, and the long half-life of the companion drug ensures the destruction of all the parasites.

New drugs are needed to be used as a combination with the standard drug treatment. The use of laboratory dyes as antimalarial agents dates back more than a century when methylene blue was used as an antimalarial by Guttman Ehrlich in 1891 [9]. There are a number of successful clinical trials *in vitro* and *in vivo* on humans in Africa of methylene blue in combination with chloroquine. This combination is now being considered seriously as a new drug due to its cost-effectiveness [10, 11].

Eosin B, another stain, has been identified as a potential antiprotozoan drug by molecular docking [12]. It has been tested *in vitro* on *Toxoplasma gondii* and on different strains of *Plasmodium falciparum* including chloroquine-resistant strains with IC50 of less than 123 nM. The reports show that its mechanism of action does not cross react with other antimalarial drugs as it targets a nonactive site on the bifunctional dihydrofolate reductase-thymidylate. Its safety has also been established in our earlier work, and the FDA has approved its use in drugs and cosmetics as it is not carcinogenic to mice when administered orally as high as 2% [13]. It has been effectively tested *in vivo* as an antimalarial agent on various kinds of mice with different routes of administration resulting in protection [14].

As the resistance shown to all three antimalarial drugs is now becoming common, an investigation was carried out to study the effect of a combination of eosin B with chloroquine or sulphadoxine-pyrimethamine or artesunate on *Plasmodium falciparum in vitro* and on *Plasmodium berghei*, a murine strain, *in vivo*.

## 2. Materials and Methods

### 2.1. Chemicals

Artesunate is a sodium salt of artemisinin (Art) which was obtained from Themis Medicare Limited (Gujarat, India) and chloroquine phosphate (CQ) from J.B. Chemicals and Pharmaceuticals Ltd. (Vatva, Ahmedabad, India). Sulphadoxine (S) and pyrimethamines (P) were obtained from Sigma-Aldrich. SP was used in the same ratio as Fansidar (20 : 1), and hence tested as one drug. All other chemicals were obtained from Merck (Germany).

### 2.2. *In Vitro* Culture

*Plasmodium falciparum* 3D7 EC No. PF3D7_0513300 clone was provided by the late Dr. Walliker, School of Tropical Medicine, London. The parasites were cultured in 7 ml of the complete RPMI 1640 medium comprising 5% serum, 10% hematocrit, 0.01 mM hypoxanthine, and 50 mg/L gentamicin (complete medium) in 75 ml flasks using the method of Trager and Jensen. The medium was changed every 48 h, and flasks were incubated at 36.5°C with 5% CO_2_, 5% O_2_, and 90% N_2_ [15].

#### 2.2.1. *In Vitro* Inhibition Assays

**Figure 1:**
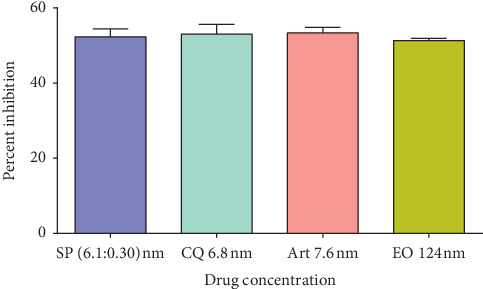
50% inhibitory concentration of antimalarial drugs on *Plasmodium falciparum in vitro*.

The 3 drugs and eosin B were diluted with the RPMI medium. The first experiment was to detect the inhibitory concentration of each drug, starting from 200 nM to 0.01 nM on *Plasmodium falciparum*, and their IC50 values were detected as EO 123 nM, SP 6.1 : 0.3 nM, CQ 6.8 nM, and Art 7.6 nM (Figure 1). The second was to obtain the same for the combination drugs. For starting concentrations, half the dosage of the IC50 values of each drug was used: EO : Art (62 nM : 3.8 nM), EO : SP (62 nM (3.05 : 0.1525 nM)), and EO : CQ (62 nM : 3.4 nM) and were serially diluted in a 96-well flat-bottomed microtiter plate with the complete culture medium. The parasite-infected erythrocytes (0.5% in 100 *μ*l) in 1.5% hematocrit and synchronized to the ring stage were added in triplicate wells. Infected erythrocytes cultured in RPMI were also used as controls. Plates were then placed in a sealed candle jar and incubated at 37°C. After 48 h, blood smears were taken from each well and stained with Giemsa for determination of parasitemia (%) by counting the number of parasitized RBCs in 5000 total RBCs. IC 30, 50, and 70 were calculated using one-way ANOVA on the GraphPad Prism software with *p* < 0.05 considered significant. Each experiment was repeated three times.

### 2.3. Animals

Healthy NMRI albino mice (18–22 g) were obtained from the animal house of the Pasteur Institute of Iran. The mice were kept under standard conditions as per the Helsinki protocol. They were provided with commercial pellet diet and water ad libitum at conditions of 22+/1 temperature and 50%–60% humidity. They were allowed to adapt to the laboratory conditions for seven days prior to the experiments. All animal work was approved by the Ethics Committee and Research Committee of the Pasteur Institute of Iran. Animals were treated as per the Helsinki Protocol for Animal Rights.

#### 2.3.1. Parasite Inoculation

*P. berghei* (ANKA strain) EC number 2.7.11.22 was kindly provided by the School of Public Health, Tehran University, Tehran, Iran. Parasites were stored at −70°C in the Elsevier's solution (2.33% glucose, 0.525% NaCl, and 1% sodium citrate in deionized water) and glycerol (9 : 1 V/V). They were grown in NMRI mice by an initial inoculum of 2 × 10^7^*P. berghei*-infected erythrocytes/ml and then sequential passaging from infected to noninfected mice weekly. Mice which were infected with (20–30%) parasitemia were used as donors. Blood was collected by heart puncture under anesthesia in tubes containing 0.5% trisodium citrate. The collected blood was diluted with normal saline so that 1 ml contains 2 × 10^7^*P. berghei*-infected RBCs, and 0.5 ml was injected into each mouse intraperitoneally (ip).

#### 2.3.2. Grouping and Dosing of Animals

For every drug, the infected mice were divided into six groups of five mice each. One group was injected by the standard drug, another by eosin as the positive control and one more as the negative control with the drug vehicle. The other three groups were given three doses of eosin (a low, medium, and high dose) in combination with a fixed dose of the concerned drug.

Eosin B was given as 200 mg/ml as the positive control and three doses of 10 mg/ml, 5 mg/ml, and 2.5 mg/ml in combination with each drug. Art was administered at 60 mg/kg as the positive control and 1.5 mg/kg in combination with the doses of eosin. CQ was given at 5 mg/kg as the positive control and combined at 0.5 mg/kg with eosin. SP was injected as 25 mg/kg : 1.25 mg/kg (in the ratio of Fansidar) as the positive control and combined with eosin at 1.5 : 0.075 mg/kg. These doses were arrived after preliminary experiments (data unpublished) in our laboratory.

### 2.4. Modified Peter's 4-Day Suppressive Test

Treatment with the drugs was carried out 2 h after infection with 0.5 ml of ip administration of 2 × 10^7^*P. berghei* parasitized erythrocytes on day 0. The mice were treated with the single drugs and combinations with eosin for 3 days every 24 h. Giemsa-stained tail blood slides were taken daily from the 3rd day onwards until the 10th day, and percent reduction of parasitemia was calculated as follows.

Differences in parasitemia percentage between treated and untreated animals were analyzed by one-way ANOVA using SPSS, and differences were considered significant if *p* < 0.05. Furthermore, the difference between the mean value of the control group (taken as 100%) and those of the experimental groups was expressed as percent reduction activity using the following equation [16]:

(1) $$\begin{array}{c} \text{percent reduction activity}=100-\left[\frac{\text{mean parasitemia treated}}{\text{mean parasitemia control}}\times 100\right]. \end{array}$$

The additive effect was calculated by the method of Chou-Talalay [17]. Isobolograms were drawn on Microsoft Excel using the IC30, 50, and 70 values of treatment with EO, CQ, Art, and SP and their combinations of the *in vitro* assay. IC30, 50, and 70 values of EO and each drug were marked on the *x* and the *y* axis, respectively, and a line which represents the additive effect was drawn between each inhibitory concentration (IC). The combination index (CI) of each treatment was calculated according to the classic isobologram equation which is the sum of the reduction index of the two drugs, where *D* is the original dose of the drug and Dx the reduced dose:

(2) $$\begin{array}{c} \text{combination index }\left(\text{CI}\right)=\left[\frac{\left(D\right)1}{\left(Dx\right)1}\right]+\left[\frac{\left(D\right)2}{\left(\text{Dx}\right)2}\right], \end{array}$$

as described previously.

CI = 1 is the additive effect, CI < 1 shows synergy, and CI > 1 exhibits antagonism. Values closer to zero show better synergy. This is depicted in the graph as the area on the right side of each IC additive line as the antagonistic effect and the left side represents synergistic effect.

## 3. Statistical Analysis

Significance was calculated at *p* < 0.05 by ANOVA in GraphPad prism software.

## 4. Results

### 4.1. Inhibition of Parasitemia by *In Vitro* Tests

**Figure 2:**
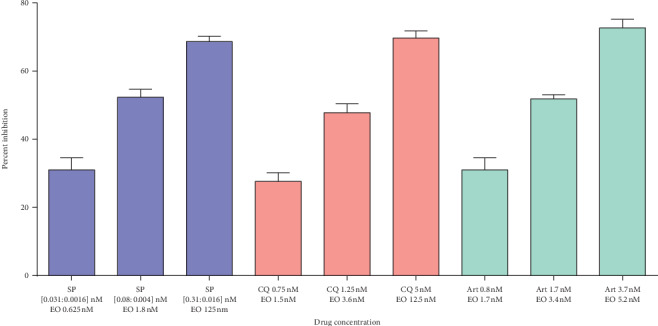
Inhibitory concentration of combination drugs on *Plasmodium falciparum in vitro.*

Inhibitory concentrations of combination drugs on *Plasmodium falciparum in vitro* and IC30, 50, and 70 of each drug singly was 124 nM for eosin B, 7.6 nM for artesunate, 6.1 : 0.1525 nM for sulphadoxine-pyrimethamine, and 6.8 nM for chloroquine (Figure 1). For the combination drugs, the IC30, IC50, and IC70 are depicted in Figure 2. As compared to the single drugs, the IC50 of the combination of artesunate : eosin shows a reduction of 4 times and 36 times than that of the dose of the positive controls. IC50 doses of chloroquine : eosin B combination also decreased to 5.4 times and 36 times. The dose for IC50 with sulphadoxine-pyrimethamine : eosin lessened to more than 75 times than that of the single drugs.

**Figure 3:**
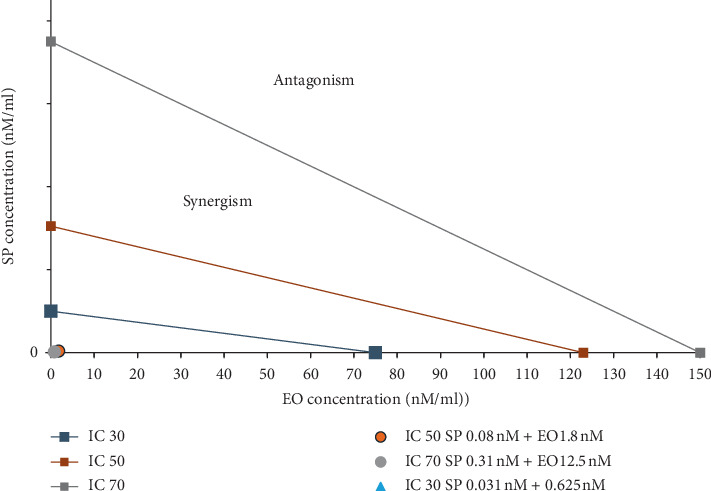
Isobologram of SP + EO combinations of *Plasmodium falciparum in vitro*. Combination index CI = 1, which is the additive effect, synergy displayed by CI < 1 and antagonism with >1, and values nearer to zero indicate better synergy. The left side of the IC additive lines shows synergy, and the right side shows antagonism of the two drugs.

Figure 3 shows the combination index of the drugs eosin and sulphadoxine-pyrimethamine. As mentioned previously, the IC30, IC50, and IC70 values of the single drugs eosin B and sulphadoxine-pyrimethamine were marked on the *x* and *y* axis with the additive lines between them and their combinations doses marked in the graph area. If the combination dose of each inhibitory concentration fall to the left of the additive lines synergy and to the right, it displays antagonism, whereas values closer to zero demonstrate better synergy. The dots in all the three doses in Figure 3 were very close to zero and hence exhibited good synergy.

**Figure 4:**
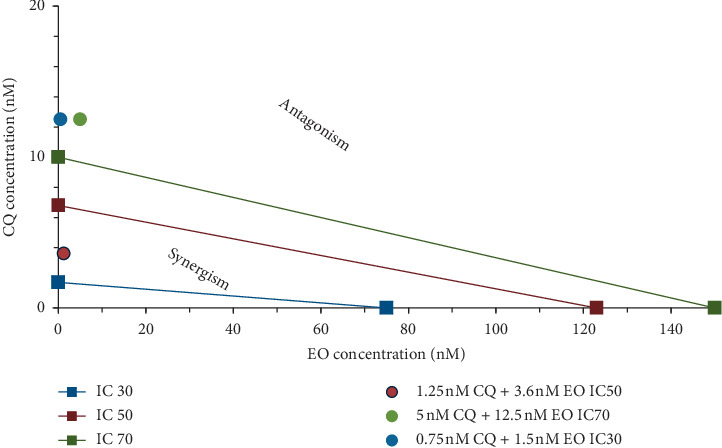
Isobologram of CQ + EO combinations of *Plasmodium falciparum in vitro*. Combination index CI = 1 which is the additive effect, synergy displayed by CI < 1 and antagonism with >1, and values nearer to zero indicate better synergy. The left side of the IC additive lines shows synergy, and the right side shows antagonism of the two drugs.

**Figure 5:**
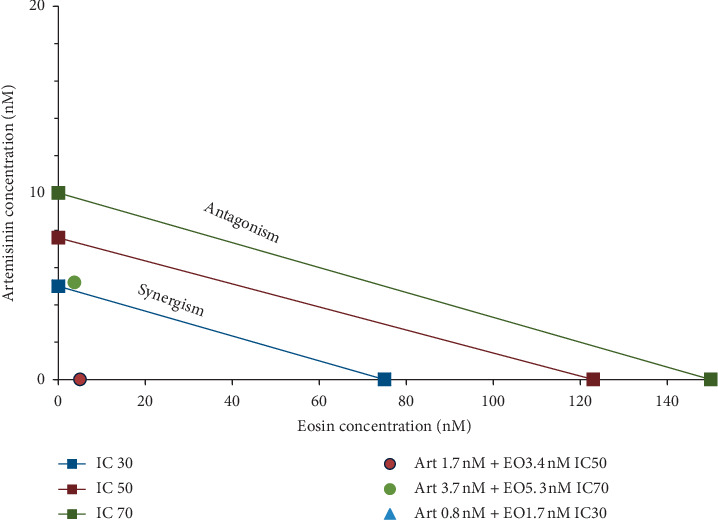
Isobologram of Art + EO combinations of *Plasmodium falciparum in vitro.* Combination index CI = 1 which is the additive effect, synergy displayed by CI < 1 and antagonism with CI > 1, and values nearer to zero indicate better synergy. The left side of the IC additive lines shows synergy, and the right side shows antagonism of the two drugs.

Figure 4 shows the combination index for chloroquine and eosin B. The two drugs show synergy only at IC50 (left of the additive line) but exhibit slight antagonism at IC30 and IC70 as observed by the position of the dose values falling to the right of the additive lines. The combination index for IC50 dose of chloroquine too is not close to zero.  Figure 5 is the isobologram depicting the eosin B : artesunate which shows very good synergy at all the doses. IC50 dose and IC30 doses of eosin B : artesunate are very close to zero, and the IC70 too exhibits synergy. IC30 is so close to zero that it cannot be seen in the graph.

### 4.2. Inhibition of Parasitemia by *In Vivo* Tests

**Figure 6:**
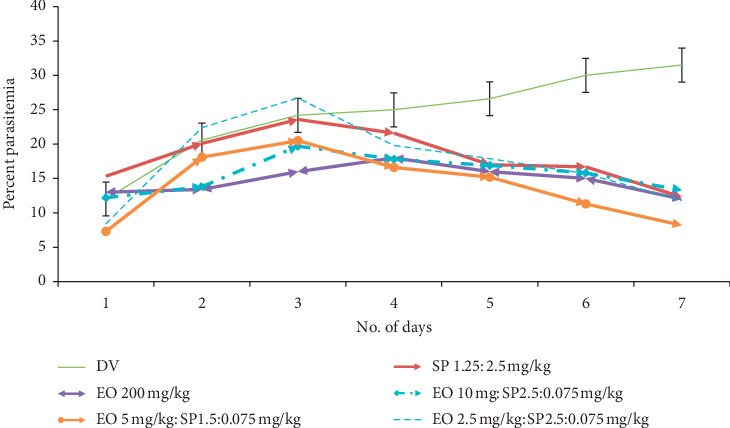
Percent parasitemia of combination of SP : EO in mice infected with *Plasmodium berghei*. Lowest percent parasitemia is demonstrated in the medium combination dose.

**Figure 7:**
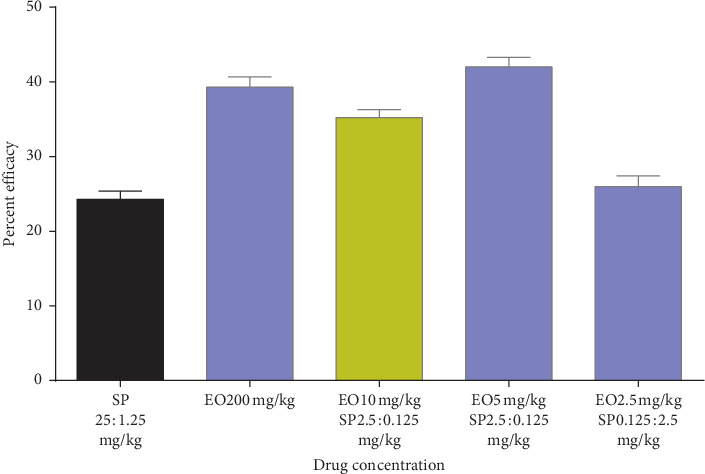
Percent efficacy of combination drug SP + EO on mice infected with *Plasmodium berghei*. The medium dose has the best effect.

**Figure 8:**
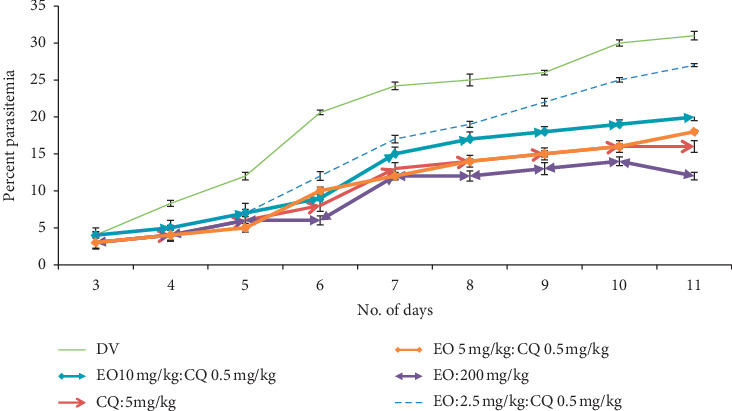
Percent parasitemia with combination drug CQ + EO on mice infected with *Plasmodium berghei*. Lowest percent parasitemia is demonstrated in the medium combination dose.

**Figure 9:**
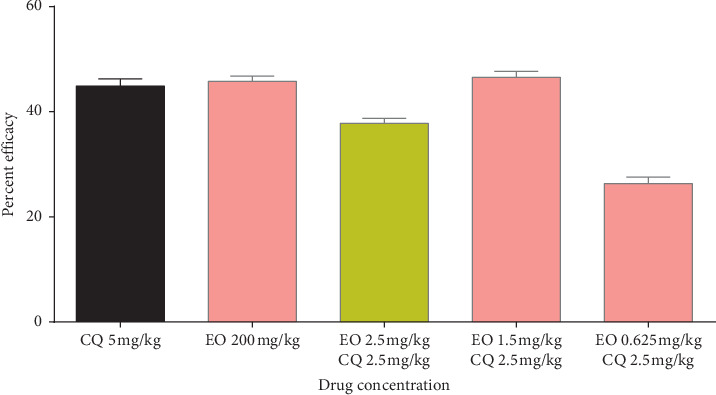
Percent efficacy of combination drug CQ + EO on mice infected with *Plasmodium berghei*. The medium dose has a moderate effect.

**Figure 10:**
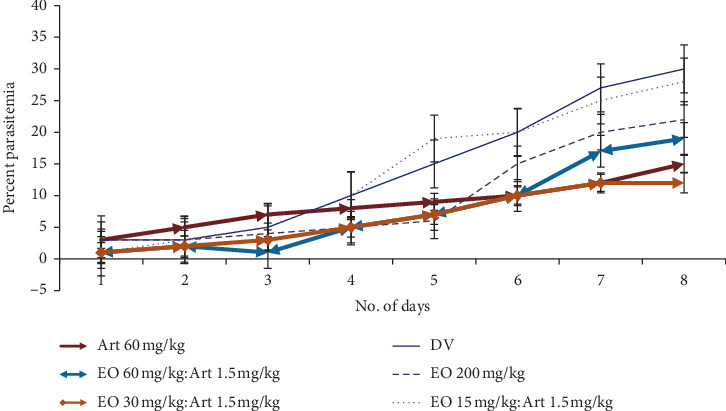
Percent parasitemia with combination drug Art + EO on mice infected with *Plasmodium berghei*. Lowest percent parasitemia is demonstrated in the medium combination dose.

**Figure 11:**
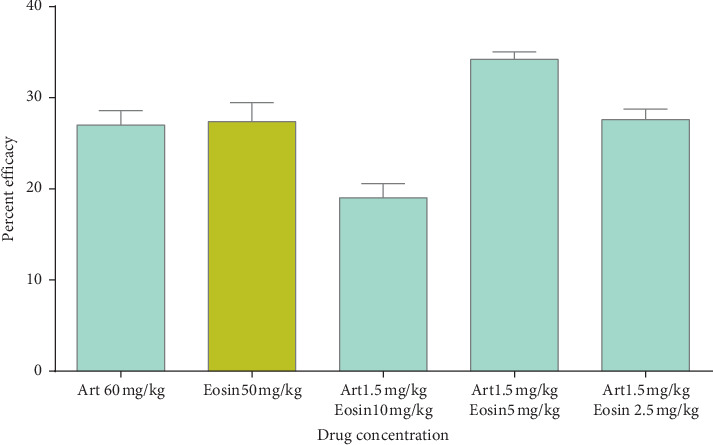
Percent efficacy of combination drug Art + EO on mice infected with *Plasmodium berghei*. The medium dose has the best effect.

Peter's suppression test was performed with the combination drugs keeping the doses of the standard drugs constant and reducing the eosin B in three doses: low, medium, and high. Percent parasitemia was counted for 10 days, and the medium doses gave the best results in all. In the eosin B : sulphadoxine pyrimethamine combination, a 10-time reduction of both the drugs gave the best inhibition where eosin B :  sulphadoxine pyrimethamine amounts were reduced to 20 : 1.5 : 0.075 mg/kg as compared with eosin B 200 mg/kg and SP 12.5 : 1.25 mg/kg, respectively, Figure 6. The percent efficacy of the eosin B : sulphadoxine-pyrimethamine combination is seen in Figure 7. Eosin B : chloroquine combination *in vivo* also showed the best results with 5 : 0.5 mg/kg compared to the standard drug eosin B 200 mg/kg and chloroquine 5 mg/kg, demonstrating a 40 times and 10 times lowering of the amount of the drugs, Figure 8. Percent efficacy of the combination of eosin B and chloroquine is shown in Figure 9. Artesunate and eosin B combination is shown in Figure 10, and its efficacy depicted in Figure 11 indicates that the medium dose had the best effect where it was lowered to 6.6 times and 20 times lesser than the dose of the single drugs with eosin B : artesunate 30 : 3 mg/kg as compared to eosin B 200 mg/kg and artesunate 60 mg/kg singly.

## 5. Discussion

Eosin B is a laboratory dye, but its safety has been approved by the FDA. No toxicity of eosin B has been demonstrated when mice were fed up to 2% of it in their diet. A single dose or even intermittent doses of eosin B does not interfere with blastocyst development in mouse embryos, and doses up to 150 nM were nontoxic to Hela cells *in vitro*[13]. Eosin B has recently been shown to be have a gametocidal effect on *Plasmodium falciparum in vitro* [18].

We tested eosin B in combination with the standard antimalarial drugs *in vitro* on *Plasmodium falciparum* and *in vivo* on mice infected with *Plasmodium berghei*. A good synergistic effect was observed with sulphadoxine-pyrimethamine. The mechanism of action of sulphadoxine-pyrimethamine is to inhibit the action of two enzymes which are important in the parasite's folate biosynthetic pathway, dihydrofolate reductase (DHFR) and dihydropteroate synthase (DHPS). Resistance is rendered by point mutations in the *DHFR* and *DHPS* genes to pyrimethamine and sulphadoxine, respectively, with *in vitro* susceptibility decreasing in *Plasmodium falciparum* related to the number of mutations in each gene [3, 19].

Sulphadoxine-pyrimethamine has been used to treat malaria in West Asia since 2004 with various responses. In Afghanistan, even after 10 years of use, there are reports of sulphadoxine-pyrimethamine sustaining its efficacy. However, there were accounts of the mutation in Iran in 2010 three years after the use of sulphadoxine-pyrimethamine as a combination drug even though sulphadoxine-pyrimethamine has not been used as monotherapy, but had been utilized in combination with chloroquine for two years which was then later replaced with sulphadoxine-pyrimethamine-artemisinin. A high number of mutations of DHFR have been detected, but as this does not correlate with clinical failure to sulphadoxine : pyrimethamine, it may have been associated with prior exposure to primaquine. However, monotherapy of sulphadoxine-pyrimethamine was followed by clinical failure in other regions; hence, combination therapies are advised by the WHO, especially those drugs which are associated with different metabolic pathways in order to overcome resistance [20, 21].

Sulphadoxine-pyrimethamine combination with eosin *in vitro* showed a highly reduced dosage in all three inhibitory concentrations (Figures 2 and 3). *In vivo* tests also decreased the effective dose to tenfold lower than the dose of the drugs singly, which is due to the fact that this combination consists of three drugs (Figures 6 and 7), but as sulphadoxine-pyrimethamine is tested in the proportion of the Fansidar drug and the ratio of the two has been kept constant, it can be treated as a single drug and its isobologram showed a very significant lowering of 50% inhibitory concentration. Its medium dose combination was seen to be the most effective in *in vivo.* It is noteworthy that pyrimethamine inhibits the activity of DHFR, and the sulfonamides and sulfones (such as sulfadoxine) inhibit the activity of dihydropteroate synthase (DHPS). Eosin B also has an effect on the folate pathway, but via the bifunctional dihydrofolate reductase-thymidylate synthase (DHFR-TS) enzyme which is unique and not present in the mammalian pathway. Eosin also acts on glutathione reductase and thioredoxin reductase, which could be one of the reasons for its additive action [13]. The combination of sulphadoxine-pyrimethamine with eosin B seems to be promising, but more work is needed on sulphadoxine-pyrimethamine-resistant *Plasmodium falciparum* strains before any firm conclusion.

Chloroquine resistance was seen in South East Asia and South America in the late 1950s. Since then, it has spread all over Africa and in most tropical countries. This drug has been discontinued in many areas for many years, but it is interesting that in Malawi where the drug was stopped for over 10 years, there was a complete disappearance of the resistant strain [22]. The persistent benefit of chloroquine to individuals with partial malaria immunity emphasizes the great need for cheap and effective drug replacement. The high cost of new antimalarials has made chloroquine as the first-line drug in individuals with symptoms of uncomplicated malaria, even where resistance is prevalent. Another laboratory dye studied is methylene blue where clinical trials in combination with chloroquine demonstrated less treatment failures than that when chloroquine was used singly [23].

It has taken many years for chloroquine resistance to develop in certain foci as it is seen to be due to multiple eight point mutations for the gene product of PfCRT which is involved in drug flux and/or pH regulation. This is unlike the resistance to pyrimethamine which arose rapidly on many independent occasions due to mutations in the dihydrofolate reductase (DHFR) gene [24].

Eosin B has demonstrated antimalarial activity on chloroquine-resistant strains of *Plasmodium falciparum* [13]. It acts on a different mechanism other than that of chloroquine, and although there was a synergy at IC50 which demonstrated a 30 time lowering of eosin and 5 times of chloroquine, there was a moderate antagonism at IC70 and IC30 (Figure 4). The *in vivo* tests also showed that none of the combination doses could give more inhibition than the single drugs (Figures 8 and 9); however, further work will have to be done to clarify these results.

Artemisinin is the drug of choice for malaria in the combination form, but due to rising resistance in South East Asia, research is on to combine artemisinin with other herbal extracts such as curcumin, piperine from pepper, and garlic (*Allium sativum)*, in the hope that these new combinations can prevent the emergence of resistance [25, 26]. It is seen that artemisinin is an inhibitor of the SE sarco/endoplasmic reticulum Ca^2+^-ATPase (SERCA) of the malarial parasite. The *Pf*ATP6 is the only SERCA-type Ca^2+^-ATPase enzyme detected in the *Plasmodium* malarial parasite and is considered to be the suitable molecular target for artemisinin. The resistance of the parasite to artemisinin could be due to mutation in the amino acid position 263 of the *Pf*ATP6 enzyme which is seen to affect the sensitivity of the enzyme to artemisinin and is located in the artemisinin binding pocket [27]. Eosin B acts on the DHFR-TS system and glutathione reductase and glutathione transferase [13]; hence, the mechanisms of action of eosin B and artemisinin on *Plasmodium* are very different, and as expected, the combination of eosin B with artesunate showed the best synergy at all three concentrations with very hopeful results. The IC50 *in vitro* tests with artesunate gave a 4.5 fold and eosin a 36 fold reduction in dosage on *Plasmodium falciparum* (Figure 5). Tests on murine malaria decreased the artesunate dose by 10 fold and eosin B by 6 fold (Figures 10 and 11); hence, this would be a good combination as their modes of action are different and a long time would be needed for emergence of drug resistance.

Eosin B in these early tests has shown very good synergy with two of the drugs, sulphadoxine-pyrimethamine and artesunate and fair synergy with chloroquine. Further work will have to be done *in vivo* to prove that the combination of eosin B and artemisinin or eosin B and sulphadoxine-pyrimethamine is beneficial. It can be concluded that eosin B provides an interesting moiety as a lead drug for further work in combination with artemisinin and other antimalarial medicines.

## 6. Conclusion

Eosin B has been tested with antimalarial drugs like chloroquine, artesunate, and sulphadoxine-pyrimethamine. Eosin B exhibited synergistic properties with artesunate, sulphadoxine-pyrimethamine, and to a certain extent with chloroquine. It may be looked upon as a candidate in the future for combination with antimalarial drugs, especially artemisinin.

## Data Availability

The data used to support the findings of this study are available from the corresponding author upon request.

## Abbreviations

EO: Eosin B
Art: Artesunate
CQ: Chloroquine
SP: Sulphadoxine-pyrimethamine
DV: Drug vehicle
CI: Combination index
IC: Inhibitory concentration.
